# Tongue feature recognition to monitor rehabilitation: deep neural network with visual attention mechanism

**DOI:** 10.3389/fbioe.2024.1392513

**Published:** 2024-05-09

**Authors:** Zhengheng Yi, Xinsheng Lai, Aining Sun, Senlin Fang

**Affiliations:** ^1^ Shenzhen Fuyong People’s Hospital, Shenzhen, China; ^2^ Guangzhou University of Chinese Medicine, Guangzhou, China; ^3^ National Famous Traditional Chinese Medicine Expert LAI Xin-sheng Inheritance Studio, Guangzhou, China; ^4^ Guangdong Zhengyuanchun Traditional Chinese Medicine Clinic Co., Ltd, Guangzhou, China; ^5^ Faculty of Data Science, City University of Macau, Macau, China

**Keywords:** traditional Chinese medicine, tongue feature recognition, deep neural network, attention mechanism, rehabilitation

## Abstract

**Objective:**

We endeavor to develop a novel deep learning architecture tailored specifically for the analysis and classification of tongue features, including color, shape, and coating. Unlike conventional methods based on architectures like VGG or ResNet, our proposed method aims to address the challenges arising from their extensive size, thereby mitigating the overfitting problem. Through this research, we aim to contribute to the advancement of techniques in tongue feature recognition, ultimately leading to more precise diagnoses and better patient rehabilitation in Traditional Chinese Medicine (TCM).

**Methods:**

In this study, we introduce TGANet (Tongue Feature Attention Network) to enhance model performance. TGANet utilizes the initial five convolutional blocks of pre-trained VGG16 as the backbone and integrates an attention mechanism into this backbone. The integration of the attention mechanism aims to mimic human cognitive attention, emphasizing model weights on pivotal regions of the image. During the learning process, the allocation of attention weights facilitates the interpretation of causal relationships in the model’s decision-making.

**Results:**

Experimental results demonstrate that TGANet outperforms baseline models, including VGG16, ResNet18, and TSC-WNet, in terms of accuracy, precision, F1 score, and AUC metrics. Additionally, TGANet provides a more intuitive and meaningful understanding of tongue feature classification models through the visualization of attention weights.

**Conclusion:**

In conclusion, TGANet presents an effective approach to tongue feature classification, addressing challenges associated with model size and overfitting. By leveraging the attention mechanism and pre-trained VGG16 backbone, TGANet achieves superior performance metrics and enhances the interpretability of the model’s decision-making process. The visualization of attention weights contributes to a more intuitive understanding of the classification process, making TGANet a promising tool in tongue diagnosis and rehabilitation.

## 1 Introduction

Traditional Chinese Medicine (TCM) practitioners monitor the rehabilitation process by carefully observing and analyzing the patient’s tongue. This method not only aids in determining the progression of the illness but also provides crucial clues for rehabilitation Du et al. (2024). Tongue diagnosis plays a pivotal role in the rehabilitation process as changes in the tongue can reflect the overall health condition of the patient. By monitoring features such as the color, shape, and moisture of the tongue, TCM practitioners can assess the progress of the patient’s rehabilitation and adjust treatment plans accordingly. Tongue features such as color, shape, and coating can be utilized to determine if a patient has an underlying health condition. Traditional Chinese tongue diagnosis Solos and Liang (2018) typically involves observations in the following aspects: 1. Tongue color: Different tongue colors may indicate various health issues. For example, a pale red tongue is often associated with good health, while a deep red tongue may suggest insufficiency of vital energy and blood; 2. Tongue shape: The shape of the tongue can also provide information about the patient’s health. For instance, an excessively large tongue, known as a fat and enlarged tongue, often accompanied by tooth imprints, may indicate the insufficiency of both the spleen and the kidney; 3. Tongue coating: The tongue coating, a thin layer of film on the tongue surface, is closely related to the intensity of dampness heat syndrome in TCM theory. Medical studies have shown a correlation between greasy tongue coating and various diseases, such as gastrointestinal disorders, and more recently, the novel coronavirus disease (COVID-19) Pang et al. (2020).

Traditional Chinese tongue diagnosis heavily relies on the subjective judgment and clinical experience of TCM practitioners, resulting in outcomes that lack objective indicators Miao et al. (2023). The adoption of computer-aided tongue feature recognition models allows for an objective and quantitative diagnosis of tongue conditions, establishing a quantifiable relationship between tongue features and diseases Zhang et al. (2006). With significant advancements in computer vision (CV), research on automatic tongue diagnosis systems based on image processing and feature recognition has become more prevalent. For instance, Zhang et al. (2015) extracted 20 color features and 20 texture features from tongue diagnosis images, including energy, entropy, contrast, and correlation, primarily describing tongue color and coating thickness. Qi et al. (2016) classified four different tongue colors, employing the ICC profile method for color correction to enhance image consistency. Subsequently, support vector machine (SVM) and random forest (RF) were employed for classification. Pang et al. (2004) introduced a computerized tongue diagnosis method based on a Bayesian network classifier, focusing on quantitative analysis of tongue color and texture features for diagnostic purposes. Song (2020) proposed a cascade classifier based on Local Binary Pattern (LBP) features to address the issue of irrelevant information interference, such as lips and cheeks in traditional Chinese tongue diagnosis images. This method utilized LBP features to describe tongue texture and employed the AdaBoost algorithm to construct the cascade classifier. Yamamoto et al. (2011) utilized a hyperspectral imaging system to acquire tongue images, identifying the most clinically relevant component vectors through Principal Component Analysis (PCA), offering an alternative approach for tongue diagnosis. Additionally, Gao et al. (2007) employed image processing algorithms to extract quantitative features of the tongue, including color and texture features, and SVM was employed for tongue classification.

However, the complexity of multiple features and variations in tongue image acquisition conditions, such as environmental factors and angles, often render traditional CV algorithms ineffective Xie et al. (2021); Li D. et al. (2022). With the rapid advancement of deep learning (DL), research on automatic tongue diagnosis programs based on DL models has gained prominence. DL methods typically exhibit stronger generalization and higher feature recognition accuracy compared to traditional computer vision algorithms, circumventing the manual feature extraction drawbacks associated with traditional machine learning methods. Most DL automatic tongue diagnosis systems encompass DL models for both tongue segmentation and tongue feature recognition. Segmentation commonly utilizes models based on U-Net Huang et al. (2020), while tongue feature recognition employs pre-trained models such as ResNet or VGG Tammina (2019). For instance, Yan J. et al. (2022) aimed to distinguish different tongue textures, such as the toughness or softness of the tongue body, through the analysis of tongue image textures. They employed the DeepLab v3+ deep learning semantic segmentation model to segment the tongue image, separating the tongue from the background. Subsequently, a ResNet101-based tongue image texture classification model was constructed. Experimental results demonstrated that using ResNet101 achieved better classification performance compared to traditional tongue image texture classification methods. In another study, Yan B. et al. (2022) proposed a convolutional neural network based on semantic modeling for tongue segmentation. Different feature extraction networks (AlexNet, VGG16, ResNet18, and DenseNet101) were compared for their effectiveness in extracting tongue color features. Combining U-Net, Inception, and dilated convolutions, Wei et al. (2022) introduced a new tongue image segmentation method called IAUNet. They designed a network named TCCNet for tongue color classification, incorporating technologies such as ResNet, Inception, and Triplet-Loss. Experimental results showed that TCCNet achieved favorable results in tongue color classification, achieving higher F1-Score and mAP compared to other baselines. Lastly, Li J. et al. (2022) employed UENET for tongue segmentation, using ResNet34 as the backbone network to extract features and perform classification from tongue photos, with overall accuracy surpassing 86%. Wang et al. (2022) develop a GreasyCoatNet model based on ResNet, which can recognize and classify different degrees of tongue greasy coating.

These above researches on tongue feature classification are mostly built based on VGG or ResNet Zhuang et al. (2022); Huang et al. (2023); Li J. et al. (2022); Wang et al. (2020). However, due to its large size, VGG or ResNet demands substantial computational resources and memory. Additionally, training directly with VGG or ResNet may lead to overfitting, especially when tongue images are challenging to collect and training data is limited. Therefore, our proposed TGANet (Togue Feature Attention Network) utilizes the pretrained VGG16’s initial five convolutional blocks as the backbone. Furthermore, we integrate an attention mechanism Fukui et al. (2019) into the backbone, aiming to mimic human cognitive attention. The primary objective is to focus model weights on crucial parts of the image. For example, in tongue coating classification, the coating is usually concentrated at the root of the tongue. If the model can prioritize local features related to coating, similar to human attention, it enhances efficiency and accuracy. Moreover, the allocation of attention weights during the learning process aids in interpreting causal relationships in the model’s judgments. Our proposed architecture TGANet is primarily based on the foundation of Yan et al. (2019).

## 2 Methods

**FIGURE 1:**
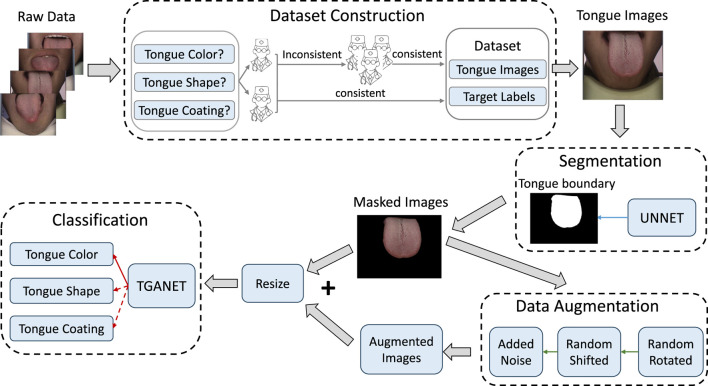
The framework for tongue color recognition encompasses four key stages: Dataset Construction, Segmentation, Data Augmentation, and Classification.

The overall framework for classifying tongue features classification is illustrated in Figure 1. Initially, the U-Net is employed to segment the input tongue images to obtain the tongue boundary. Subsequently, the masked image derived from the tongue boundary is followed by a data augmentation process. Specifically, the masked image undergoes sequential random rotation, shifting, and adding noise. Following this augmentation, both the masked images and their augmented counterparts are fed into the TGANet to execute the classification of tongue features. In the classification phrase, three kinds of tongue features are classified: tongue color, tongue shape, and tongue coating.

### 2.1 Dataset Construction

The publicly available BioHit image dataset comprises 300 tongue images with dimensions of 567 × 768 pixels. We annotated this original dataset with diagnostic labels. The image annotation process involves three steps. Firstly, domain experts engaged in discussions to establish diagnostic criteria for each category within the three types of tongue features. The detail of the three categories and their class labels is shown in Table 1. Subsequently, two well-trained TCM practitioners from the Guangzhou University of Chinese Medicine independently assessed each tongue image to distinguish the class labels for each tongue feature. A third TCM professional with 20 years of expertise joined the deliberations to collectively resolve any disputes and achieve a final consensus. Images with unanimous agreement were then incorporated into the dataset for the development of a deep learning-based tongue feature recognition model.

**TABLE 1:** Tongue feature labels and corresponding descriptions.

Label∖Tongue feature	Tongue color	Tongue body	Tongue coating
0	Pale Red	Swollen	White Greasy
1	Red	Non-Swollen	Thin White
2	Dark Red	N/A	Thin Yellow
3	N/A	N/A	Yellow Greasy

### 2.2 Image segmentation

**FIGURE 2:**
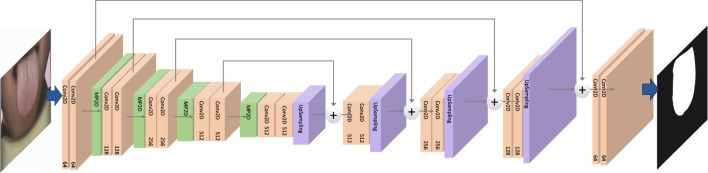
The architecture of U-Net for tongue image segmentation.

The aim of tongue image segmentation is to enhance the effectiveness of tongue feature classification by eliminating extraneous information in the image, such as interference from the human jaw or background details, which can disrupt the classification process. To achieve this, we employed the deep convolutional neural network U-Net for tongue segmentation. U-Net is widely utilized in image segmentation, drawing inspiration from semantic segmentation tasks and designed to deliver high-resolution, precise segmentation results.The overall architecture of the U-Net dedicated to segmenting the contour images of the tongue is illustrated in Figure 2. U-Net adopts an encoder-decoder structure. The encoder is responsible for sequentially extracting features from the input tongue image through convolution and pooling operations, progressively reducing spatial resolution. The decoder gradually restores spatial resolution through upsampling and deconvolution operations. U-Net incorporates skip connections by linking the output of the last convolutional layer of each encoder block to the corresponding layer in the decoder. This helps retain more detailed information at different resolutions, overcoming potential information loss in deep networks. The final segmentation output is generated in the last layer using a 1 × 1 convolutional layer. The training utilizes the cross-entropy loss function to measure the difference between the model’s output and the actual segmented image. By leveraging U-Net, we obtain the masked tongue image by acquiring the tongue segmentation contour mask from the input tongue image.

### 2.3 Image augmentation

Due to the limited number of samples in medical images and the imbalance in the number of samples for each category, data augmentation is applied to the samples before image classification. This ensures that the quantity of each category in tongue feature classification remains consistent, maintaining an equal number of samples for both the training and validation sets. The commonly employed method to balance categories involves setting the upper limit based on the category with the maximum sample count and augmenting samples from categories with fewer samples.

**FIGURE 3:**
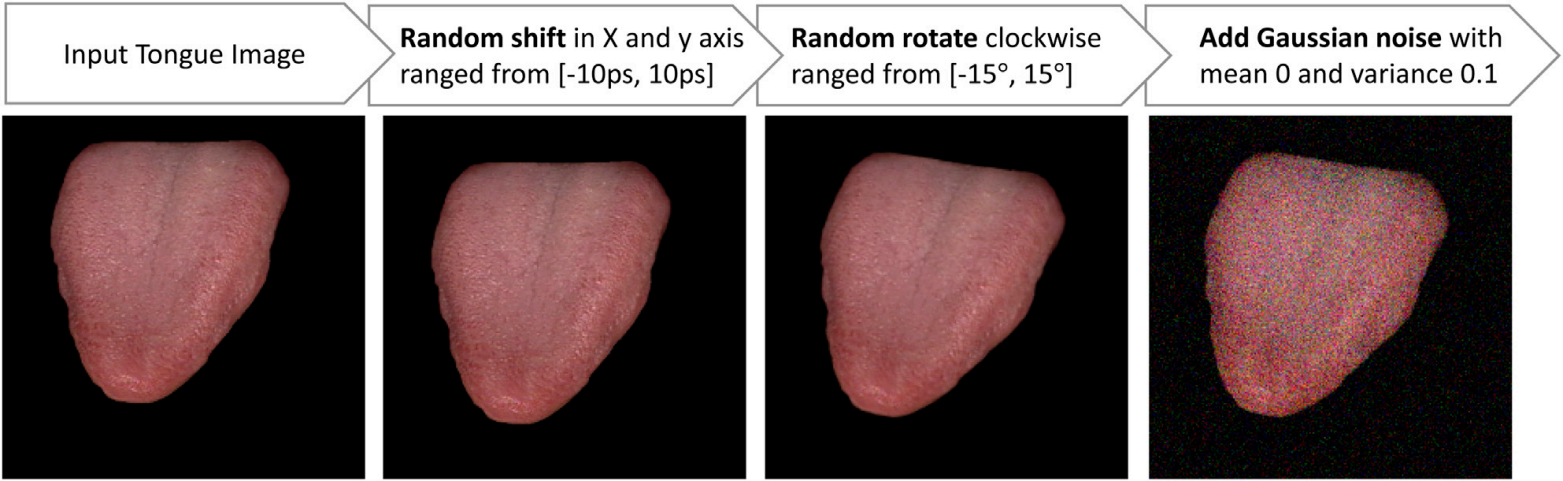
The input tongue images undergo augmentation through the following actions: random shift, random rotation, and the addition of Gaussian noise.

Various data augmentation techniques are typically utilized, including random translation, random rotation, and the addition of Gaussian noise in different combinations to enhance images. As shown in Figure 3, we implemented the augmentation in the order of random translation, followed by random rotation, and then the addition of Gaussian noise. Specifically, random translation involves random shifts in both the *x* and *y*-axes within the range from −10 pixels (ps) to 10 ps. Random rotation includes clockwise rotation within the range from −15° to 15°, and Gaussian noise is added with a mean of 0 and a variance of 0.1.

### 2.4 Tongue feature classification

**FIGURE 4:**
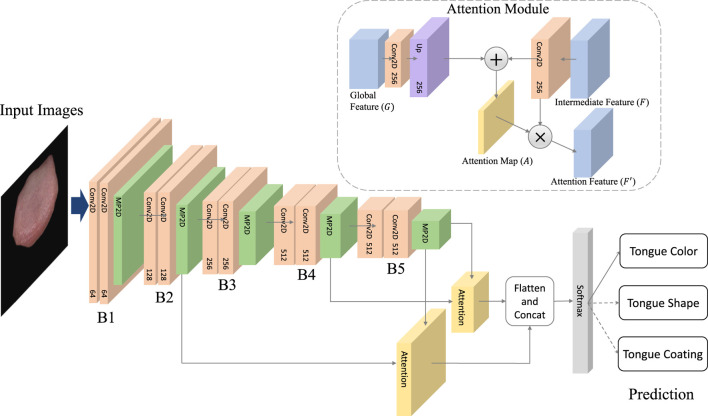
The architecture of TGANet.The TGANet employs the VGG16 architecture as its backbone, with all fully connected layers removed. Input images undergo sequential processing through convolutional blocks *B*1 to *B*5, capturing global features from the input data. Intermediate features (denoted as *F*) extracted from pooling layers in *B*2 and *B*4 are utilized for attention map learning. Additionally, the output of the pooling layer following *B*5 (denoted as *G*) represents the global features aggregated by all convolutional blocks within the network.

The overall architecture of the TGANet model for tongue feature classification is illustrated in Figure 4. We employ VGG16 as the model’s backbone, removing all fully connected layers. Input images sequentially pass through convolutional blocks *B*1 to *B*5, extracting global features from the input images. Intermediate features (denoted as *F*) obtained from pooling layers in *B*2 and *B*4 are used to learn attention maps, while the output of the pooling layer after *B*5 (denoted as *G*) represents global features extracted by all convolutional blocks in the network. Intermediate feature *F* and global feature *G* are jointly input into the Attention Module to obtain attention feature *F*:

$$F^{\prime }=Attention\left(F,G\right).$$ (1)

Here, “Attention” represents the operation within the attention module. Specifically, to match the sizes of intermediate and global features, *F* undergoes a convolutional layer to increase its channel count to 256, and bilinear interpolation aligns its feature size with *G*. *G* undergoes a convolutional layer to compress its channel count to 256. The transformed *F* and *G* are then added to obtain *U*:

$$U=W_{F}*F+UP\left(W_{G}*G\right).$$ (2)

The * symbol denotes convolutional operation, and *UP* represents bilinear interpolation. *W*_*F*_ and *W*_*G*_ are the convolutional weights for *F* and *G*, respectively. Next, *U* undergoes an operation to transform into an attention map *A*:

$$A=Sigmoid\left(Conv\left(ReLU\left(U\right)\right)\right).$$ (3)

Subsequently, pixel-wise multiplication of *F* and *A* yields the Attention Feature:

$$F^{\prime }=A*F.$$ (4)

Finally, attention features generated from intermediate features (*B*2 and *B*4) are concatenated with global features. The softmax operation is applied to obtain the final predictions for tongue features. Specifically, predictions are made for three different tongue features: tongue color, tongue shape, and tongue coating. The overall architecture is trained end-to-end.

### 2.5 Model evaluation

The model training was conducted on a Windows 11 system equipped with an NVIDIA 4090 GPU, utilizing Python and PyTorch. Initial model parameters were initialized with weights pre-trained on the ImageNet dataset. This transfer learning strategy endowed the model with robust prior knowledge, contributing to superior performance. Subsequently, fine-tuning of the model parameters was performed using the tongue dataset. The parameters of the Attention module were initialized using the Kaiming initialization method, and model optimization employed the Adam optimizer with a learning rate of 0.0001. Three distinct tongue feature classifications shared the same model structure, with the only difference lying in the output of the final fully connected classification layer, which was adjusted according to the different categories. During the training process, model parameter updates were achieved by minimizing the cross-entropy loss function. All models underwent 20 training epochs with a batch size of 20, and model parameters were fixed based on the best performance observed on the validation dataset. Training and testing the model on the tongue color, shape, and coating classification respectively with the 5-fold cross-validation.

### 2.6 Metric

Accuracy is the proportion of correctly classified samples by the model on the entire dataset. In model evaluation, accuracy is a crucial metric for assessing the overall performance of the model. The calculation of accuracy *Acc* is the ratio of the number of samples correctly predicted by the model to the total number of samples:

$$Acc=\frac{N_{c}}{N_{t}},$$ (5)

where *N*_*c*_ is the number of correctly predicted samples, and *N*_*t*_ is the total number of samples.

Precision Ashley (2016) refers to the proportion of actual positive samples among all the samples predicted as positive by the model. In some applications, high precision may be a key objective as it indicates the accuracy of the model in positive class predictions. Precision *P* is calculated as:

$$P=\frac{TP}{TP+FP},$$ (6)

where *TP* represents true positives, indicating the number of samples correctly predicted as positive by the model, and *FP* represents false positives, indicating the number of instances where the model incorrectly predicted negative class samples as positive.

F1 Score Goutte and Gaussier (2005) is the harmonic mean of precision and recall, used to comprehensively consider the model’s accuracy and recall performance. In some situations, the F1 Score is used as a balance between precision and recall. The calculation of F1 Score *F*1 is given by:

$$F1=2\times \frac{P\times R}{P+R},$$ (7)

where *R* is recall, also known as sensitivity or true positive rate, is a metric that measures the ability of a model to capture all positive instances in the dataset. It is defined as the ratio of *TP* to the sum of *TP* and False Negatives (positive samples incorrectly predicted as negative). The formula for *R* is given by:

$$R=\frac{TP}{TP+FN}.$$ (8)

AUC Wu and Flach (2005) is the area under the ROC curve, where the ROC curve illustrates the trade-off between true positive and false positive rates at different thresholds. A higher AUC value, closer to 1, indicates better model performance. AUC is commonly used for performance evaluation in binary classification problems, especially when dealing with imbalanced datasets. The specific calculation of AUC is not enumerated here but is typically obtained by integrating the ROC curve.

## 3 Results

### 3.1 Baseline models

Throughout the experiments, we compare the TGANet with the following models.

1) VGG16: VGG16 Tammina (2019) is a deep convolutional neural network architecture designed for image classification tasks. The “16″ in VGG16 refers to the network’s depth. VGG16 follows a simple and uniform architecture with small 3 × 3 convolutional filters, which helps maintain a consistent receptive field. It also employs max-pooling layers for spatial down-sampling. The pre-trained VGG16 weights on large datasets ImageNet to initialize their models before fine-tuning for our tongue feature classification tasks.
2) ResNet18 Odusami et al. (2021): short for Residual Network with 18 layers, is a convolutional neural network architecture introduced by Kaiming He et al. It is part of the ResNet family, known for its deep structure and the incorporation of residual learning blocks. The architecture includes a stack of residual blocks, where each block consists of two convolutional layers with batch normalization and rectified linear unit (ReLU) activation functions. The key innovation in ResNet architectures is the use of skip connections or shortcuts that skip one or more layers, allowing the gradient to flow more easily during backpropagation. This facilitates the training of very deep networks and helps alleviate the vanishing gradient problem. ResNet18 architecture serves as a baseline model and is widely used in tongue feature classification tasks due to its effectiveness and efficiency.
3) TSC-WNet Huang et al. (2023): TSC-WNet is a comprehensive neural network architecture designed for the classification of tongue size and shape. TSC-WNet consists of two subnetworks: TSC-UNet and TSC-Net. TSC-Net serves as the classification backbone, while TSC-UNet is responsible for tongue segmentation. TSC-Net employs a simple and efficient architecture with four convolutional layers. By combining both classification and segmentation features, TSC-WNet shows the best validation accuracy and steady performance during training. TSC-WNet is a well-designed network architecture that integrates classification and segmentation tasks, showcasing improved accuracy and robust performance in the challenging domain of tongue analysis.

### 3.2 Tongue feature classification model performance

Table 2 presents a comprehensive performance comparison of various tongue classification models, including ResNet18, TS-WCNet, and our proposed TGANet. The result is the mean and standard deviation of the five folds by using 5-fold cross-validation. The models were evaluated based on different tongue features: Tongue Color, Tongue Shape, and Tongue Coating by using 5-fold cross-validation. For the Tongue Color feature, TGANet outperformed both VGG16, ResNet18, and TSC-WNet with a remarkable accuracy of 91.88%, precision of 90.53%, F1 score of 89.87%, and AUC of 96.45%. These results highlight the superior performance of TGANet in capturing color-related information for tongue classification. Similarly, when focusing on the Tongue Shape feature, TGANet demonstrated a significant improvement in accuracy (92.38%), precision (94.93%), and F1 score (94.05%) compared to VGG16, ResNet18, and TS-WCNet. The robustness of TGANet in extracting shape-related features contributes to its outstanding performance. In the case of Tongue Coating classification, TGANet exhibited outstanding results with an accuracy of 94.77%, precision of 95.59%, and F1 score of 95.02%. This emphasizes the efficacy of TGANet in recognizing and classifying diverse tongue coating patterns. Additionally, the uncertainty in the model’s performance is captured through the standard deviation, providing insights into the stability of the results across multiple evaluations. The consistent outperformance of TGANet across different tongue features underscores its robustness and effectiveness in tongue classification tasks.

**TABLE 2:** Comparison of the metrics between our proposed TGANet and the baselines on the three tongue feature classifications (Mean ± SEM). The result is the mean and standard deviation of the five folds by using 5-fold cross-validation. The best performance is marked in bold.

Model	Tongue feature	Accuracy (%)	Precision (%)	F1 score (%)	AUC (%)
VGG16	Tongue Color	75.13 ± 4.64	75.43 ± 5.03	69.16 ± 5.96	84.31 ± 3.02
ResNet18		82.37 ± 6.42	82.87 ± 5.40	80.86 ± 5.78	93.49 ± 3.10
TSC-WNet		83.08 ± 4.68	82.76 ± 4.92	79.72 ± 6.45	92.76 ± 3.59
TGANet (our)		**91.88** ± **2.65**	**90.53** ± **3.16**	**89.87** ± **3.17**	**96.45** ± **1.94**
VGG16	Tongue Shape	91.93 ± 1.70	94.15 ± 2.87	93.55 ± 3.16	96.31 ± 3.04
ResNet18		91.43 ± 2.65	91.15 ± 2.69	90.25 ± 2.89	**97.89** ± **1.47**
TSC-WNet		89.83 ± 2.00	90.88 ± 1.84	88.83 ± 2.85	94.74 ± 1.80
TGANet (our)		**92.38** ± **1.43**	**94.93** ± **1.63**	**94.05** ± **2.13**	97.55 ± 1.57
VGG16	Tongue Coating	91.69 ± 1.41	94.16 ± 2.40	93.50 ± 2.61	98.46 ± 0.70
ResNet18		90.16 ± 5.17	93.34 ± 3.67	91.92 ± 5.60	**98.80** ± **0.62**
TSC-WNet		84.62 ± 2.69	87.32 ± 3.47	85.54 ± 3.80	95.87 ± 1.63
TGANet (our)		**94.77** ± **1.02**	**95.59** ± **1.52**	**95.02** ± **1.72**	98.77 ± 1.21

### 3.3 Attention visualization

Attention Visualization is designed to add a visualization of attention weights (attention map) to input images. Initially, the input image is transformed from a PyTorch tensor to a NumPy array, with channel dimensions adjusted to the correct order. The attention map’s size is adjusted based on an upsampling factor using bilinear interpolation. The grayscale attention map is then converted to a heatmap using the JET color map from OpenCV. Finally, the image and normalized attention map are blended in a certain proportion, creating an overlay of attention visualization on the image. This process visualizes the depth of focus of a deep learning model on the input. This is particularly helpful in understanding the decision-making process of a deep learning model in tongue feature classification, emphasizing regions considered crucial for tongue segmentation tasks.

**FIGURE 5:**
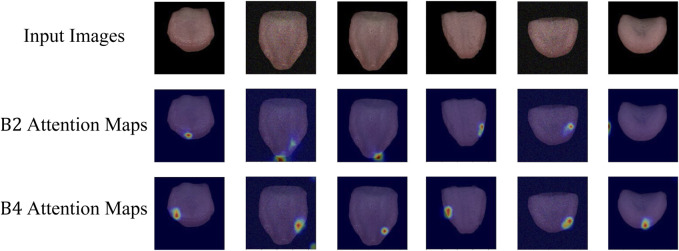
Visualization of TGANet attention weights for tongue color classification. B2 Attention Maps are the attention weights learning from the intermediate features of *B*2, and B4 Attention Maps are the attention weights learning from the intermediate features of *B*4.

**FIGURE 6:**
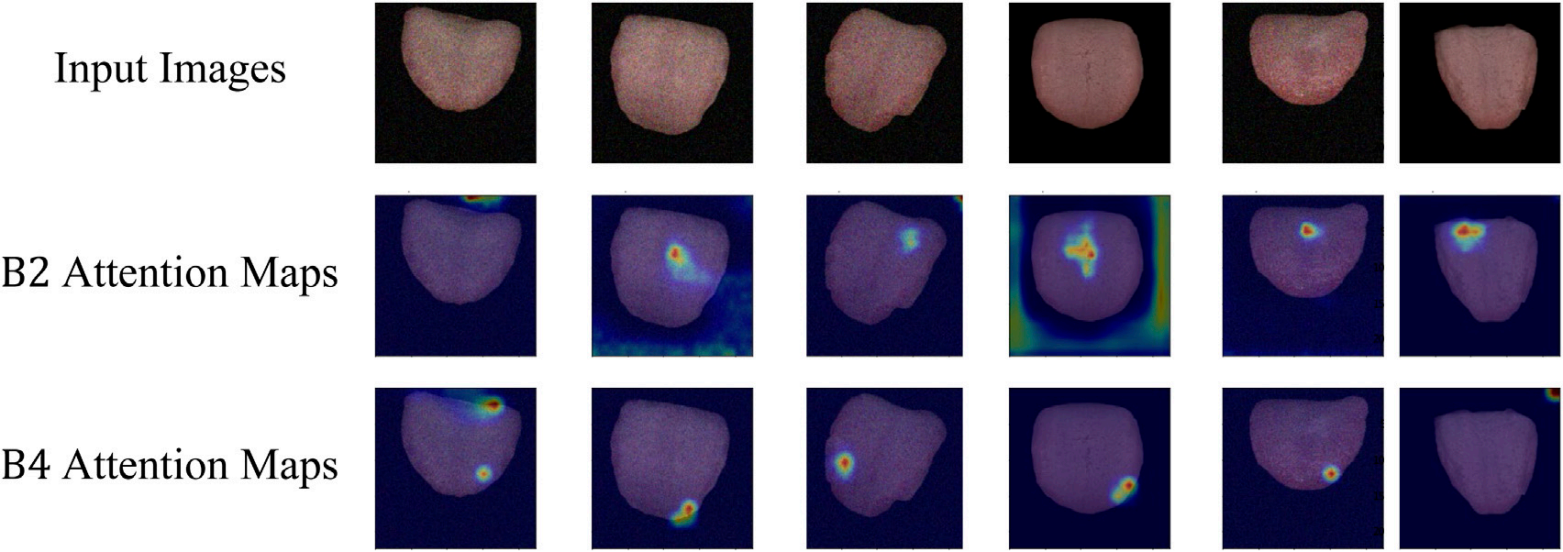
Visualization of TGANet attention weights for tongue coating classification.

**FIGURE 7:**
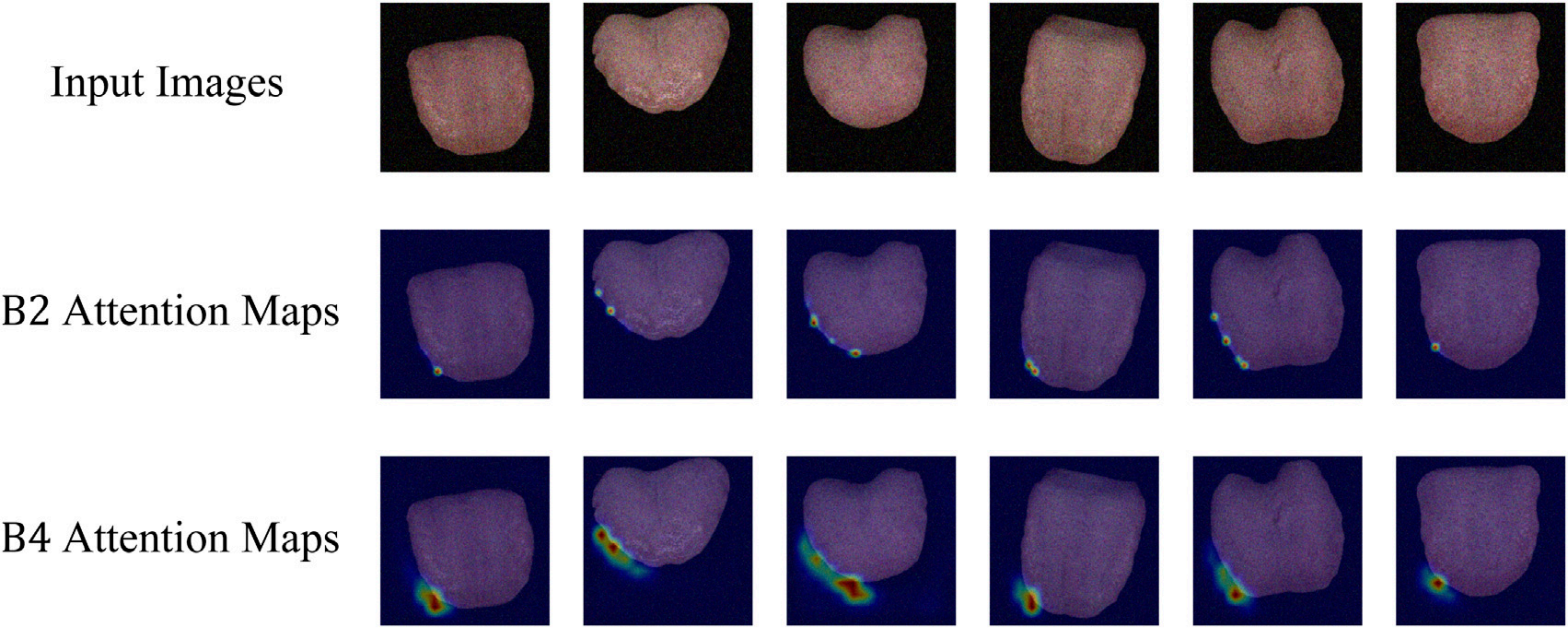
Visualization of TGANet attention weights of TGANet for tongue shape classification.

As depicted in Figure 5, the attention visualization images for tongue color classification show that the model primarily utilizes features from the tip of the tongue in its decision-making process, which is reasonable given that the color of the tongue tip is typically more distinct. As shown in Figure 6, the attention visualization images for tongue coating classification reveal that the model’s decision-making relies heavily on features from the root of the tongue, which is sensible as tongue coating is mainly concentrated in the root area. Figure 7 illustrates the attention visualization images for tongue shape classification, demonstrating that the model’s decision-making focuses on the contour features of the tongue. This aligns with the common practice among practitioners who assess the thickness and appearance of the tongue’s outline to determine its texture.

## 4 Discussion

TCM practitioners can moniter the patient rehabilitation process by carefully observing and analyzing the patient’s tongue feature. For instance, a deep red tongue may suggest a deficiency in vital energy and blood, a fat and enlarged tongue may indicate the insufficiency of both the spleen and the kidney, and a thin layer of film on the tongue surface is closely linked to the intensity of dampness-heat syndrome in TCM theory. Here, we propose a framework designed for the classification of three distinct tongue features. Initially, our expert physicians labeled a publicly available dataset, BioHit, based on these three different tongue features. Subsequently, we preprocessed and augmented the images using image segmentation and augmentation techniques. Then, employing the TGANet architecture with an attention mechanism, we classified the three different tongue features. Our TGANet model outperforms baseline models, achieving the highest accuracy, precision, F1 score, and AUC metrics.

Additionally, the TGANet, based on the VGG16 architecture with attention modules, exhibits superior performance. Compared to the VGG16 without attention modules, the attention modules in our TGANet were further visualized. It was observed that for different tongue feature classifications, the neural network’s attention weights varied. For tongue color classification, attention weights were concentrated on the tongue tip; for tongue shape classification, attention weights were focused on the tongue contour; for tongue coating classification, attention weights were centered around the tongue base. This alignment with the expertise of physicians emphasizes the effectiveness of the features learned by our model. Furthermore, the visualization of attention modules provides interpretability for deep learning-based tongue diagnostic models.

In practical applications, establishing a universal model applicable to various tongue feature classifications is highly meaningful in tongue diagnosis and rehabilitation. This contributes to mitigating the overfitting problem. As a result, our TGANet demonstrates outstanding performance in different tongue feature classifications compared to baselines, ultimately leading to more precise diagnoses and better patient rehabilitation in TCM.

## 5 Conclusion

In conclusion, our study introduces TGANet, a novel DL model designed for the classification of crucial tongue features in TCM. Leveraging the initial five convolutional blocks of pre-trained VGG16 as the backbone and integrating an Attention mechanism, TGANet outperforms baseline models in accuracy, precision, F1 score, and AUC metrics for distinguishing tongue color, coating, and shape. The integration of an attention mechanism provides interpretability by emphasizing model weights on significant regions of the tongue image. TGANet exhibits robust performance, and the visualization of attention weight further reveals the model’s focus on specific tongue regions for decision-making, aligning with clinical practices. This study contributes to advancing automatic tongue diagnosis systems, providing a foundation for objective and quantitative assessment of tongue conditions in TCM.

## Data Availability

The original contributions presented in the study are included in the article/Supplementary material, further inquiries can be directed to the corresponding author.

## References

[B1] Ashley E. A. (2016). Towards precision medicine. Nat. Rev. Genet. 17, 507–522. 10.1038/nrg.2016.86 27528417

[B2] Du T. Liu J. Xie H. Wang X. Yang X. Yang Y. (2024). Multifunctional coatings of nickel-titanium implant toward promote osseointegration after operation of bone tumor and clinical application: a review. Front. Bioeng. Biotechnol. 12, 1325707. 10.3389/fbioe.2024.1325707 38444648 PMC10912669

[B3] Fukui H. Hirakawa T. Yamashita T. Fujiyoshi H. (2019). “Attention branch network: learning of attention mechanism for visual explanation,” in Proceedings of the IEEE/CVF conference on computer vision and pattern recognition (IEEE), 10705–10714.

[B4] Gao Z. Po L. Jiang W. Zhao X. Dong H. (2007). “A novel computerized method based on support vector machine for tongue diagnosis,” in 2007 third international IEEE conference on signal-image technologies and internet-based system (IEEE), 849–854.

[B5] Goutte C. Gaussier E. (2005). “A probabilistic interpretation of precision, recall and f-score, with implication for evaluation,” in European conference on information retrieval (Springer), 345–359.

[B6] Huang H. Lin L. Tong R. Hu H. Zhang Q. Iwamoto Y. (2020). “Unet 3+: a full-scale connected unet for medical image segmentation,” in ICASSP 2020-2020 IEEE international conference on acoustics, speech and signal processing (ICASSP) (IEEE), 1055–1059.

[B7] Huang Y. Li X. Zheng S. Li Z. Li S. Shen L. (2023). Tongue size and shape classification fusing segmentation features for traditional Chinese medicine diagnosis. Neural Comput. Appl. 35, 7581–7594. 10.1007/s00521-022-08054-y

[B8] Li D. Hu J. Zhang L. Li L. Yin Q. Shi J. (2022a). Deep learning and machine intelligence: new computational modeling techniques for discovery of the combination rules and pharmacodynamic characteristics of traditional Chinese medicine. Eur. J. Pharmacol. 933, 175260. 10.1016/j.ejphar.2022.175260 36116517

[B9] Li J. Zhang Z. Zhu X. Zhao Y. Ma Y. Zang J. (2022b). Automatic classification framework of tongue feature based on convolutional neural networks. Micromachines 13, 501. 10.3390/mi13040501 35457806 PMC9025353

[B10] Miao J. Huang Y. Wang Z. Wu Z. Lv J. (2023). Image recognition of traditional Chinese medicine based on deep learning. Front. Bioeng. Biotechnol. 11, 1199803. 10.3389/fbioe.2023.1199803 37545883 PMC10402920

[B11] Odusami M. Maskeliunas R. Damaševičius R. Krilavičius T. (2021). Analysis of features of alzheimer’s disease: detection of early stage from functional brain changes in magnetic resonance images using a finetuned resnet18 network. Diagnostics 11, 1071. 10.3390/diagnostics11061071 34200832 PMC8230447

[B12] Pang B. Zhang D. Li N. Wang K. (2004). Computerized tongue diagnosis based on bayesian networks. IEEE Trans. Biomed. Eng. 51, 1803–1810. 10.1109/tbme.2004.831534 15490827

[B13] Pang W. Zhang D. Zhang J. Li N. Zheng W. Wang H. (2020). Tongue features of patients with coronavirus disease 2019: a retrospective cross-sectional study. Integr. Med. Res. 9, 100493. 10.1016/j.imr.2020.100493 32817820 PMC7424212

[B14] Qi Z. Tu L.-p. Chen J.-b. Hu X.-j. Xu J.-t. Zhang Z.-f. (2016). The classification of tongue colors with standardized acquisition and icc profile correction in traditional Chinese medicine. BioMed Res. Int. 2016, 1–9. 10.1155/2016/3510807 PMC5168476 28050555

[B15] Solos I. Liang Y. (2018). A historical evaluation of Chinese tongue diagnosis in the treatment of septicemic plague in the pre-antibiotic era, and as a new direction for revolutionary clinical research applications. J. Integr. Med. 16, 141–146. 10.1016/j.joim.2018.04.001 29691189

[B16] Song C. (2020). Tongue localization method based on cascade classifier. J. Artif. Intell. Pract. 3, 13–21. 10.23977/jaip.2020.030104

[B17] Tammina S. (2019). Transfer learning using vgg-16 with deep convolutional neural network for classifying images. Int. J. Sci. Res. Publ. (IJSRP) 9, 94200–p10150. 10.29322/ijsrp.9.10.2019.p9420

[B18] Wang X. Liu J. Wu C. Liu J. Li Q. Chen Y. (2020). Artificial intelligence in tongue diagnosis: using deep convolutional neural network for recognizing unhealthy tongue with tooth-mark. Comput. Struct. Biotechnol. J. 18, 973–980. 10.1016/j.csbj.2020.04.002 32368332 PMC7186367

[B19] Wang X. Wang X. Lou Y. Liu J. Huo S. Pang X. (2022). Constructing tongue coating recognition model using deep transfer learning to assist syndrome diagnosis and its potential in noninvasive ethnopharmacological evaluation. J. Ethnopharmacol. 285, 114905. 10.1016/j.jep.2021.114905 34896205

[B20] Wei L. Jinming C. Bo L. Wei H. Xingjin W. Hui Z. (2022). Tongue image segmentation and tongue color classification based on deep learning. Digit. Chin. Med. 5, 253–263. 10.1016/j.dcmed.2022.10.002

[B21] Wu S. Flach P. (2005). “A scored auc metric for classifier evaluation and selection,” in Second workshop on ROC analysis in ML (bonn, Germany).

[B22] Xie J. Jing C. Zhang Z. Xu J. Duan Y. Xu D. (2021). Digital tongue image analyses for health assessment. Med. Rev. 1, 172–198. 10.1515/mr-2021-0018 PMC10388765 37724302

[B23] Yamamoto S. Tsumura N. Nakaguchi T. Namiki T. Kasahara Y. Ogawa-Ochiai K. (2011). Principal component vector rotation of the tongue color spectrum to predict “mibyou”(disease-oriented state). Int. J. Comput. assisted radiology Surg. 6, 209–215. 10.1007/s11548-010-0506-8 20574797

[B24] Yan B. Zhang S. Yang Z. Su H. Zheng H. (2022a). Tongue segmentation and color classification using deep convolutional neural networks. Mathematics 10, 4286. 10.3390/math10224286

[B25] Yan J. Chen B. Guo R. Zeng M. Yan H. Xu Z. (2022b). Tongue image texture classification based on image inpainting and convolutional neural network. Comput. Math. Methods Med. 2022, 1–11. 10.1155/2022/6066640 PMC9780003 36570335

[B26] Yan Y. Kawahara J. Hamarneh G. (2019). “Melanoma recognition via visual attention,” in Information Processing in Medical Imaging: 26th International Conference, IPMI 2019, Hong Kong, China, June 2–7, 2019 (Springer), 793–804.

[B27] Zhang H. Wang K. Zhang D. Pang B. Huang B. (2006). “Computer aided tongue diagnosis system,” in 2005 IEEE engineering in medicine and biology 27th annual conference (IEEE), 6754–6757. 10.1109/IEMBS.2005.1616055 17281824

[B28] Zhang X. Zhang J. Hu G. Wang Y. (2015). “Preliminary study of tongue image classification based on multi-label learning,” in Proceedings, Part III 11 Advanced Intelligent Computing Theories and Applications: 11th International Conference, ICIC 2015, Fuzhou, China, August 20-23, 2015 (Springer), 208–220.

[B29] Zhuang Q. Gan S. Zhang L. (2022). Human-computer interaction based health diagnostics using resnet34 for tongue image classification. Comput. Methods Programs Biomed. 226, 107096. 10.1016/j.cmpb.2022.107096 36191350

